# Long-Term Outcomes of EUS-Guided Pancreatic Duct Drainage for the Management of Benign Pancreaticojejunostomy Anastomotic Stricture—A Retrospective Cohort Study

**DOI:** 10.3390/jcm14238439

**Published:** 2025-11-28

**Authors:** Indria Melianti, Kazuo Hara, Takamichi Kuwahara, Shin Haba, Nozomi Okuno, Shimpei Matsumoto, Hiroki Koda, Keigo Oshiro, Tomoki Ogata

**Affiliations:** Department of Gastroenterology, Aichi Cancer Center Hospital, Nagoya 464-8681, Japan; meliantiindria@gmail.com (I.M.); kuwa_tak@aichi-cc.jp (T.K.); s.haba@aichi-cc.jp (S.H.); nokuno@aichi-cc.jp (N.O.); s.matsumoto@aichi-cc.jp (S.M.); h.koda@aichi-cc.jp (H.K.); k.oshiro@aichi-cc.jp (K.O.); tomo1993baske@gmail.com (T.O.)

**Keywords:** endosonography, pancreatic ducts, pancreaticoduodenectomy

## Abstract

**Background/Objectives:** Endoscopic ultrasound-guided pancreatic duct drainage (EUS-guided PDD) has emerged as an important technique for managing pancreaticojejunostomy anastomotic strictures (PJAS) following pancreatoduodenectomy. However, long-term outcome data remain limited. This study aimed to evaluate the long-term outcomes of EUS-guided PDD for benign PJAS. **Methods:** This single-center retrospective cohort study included 46 patients who underwent technically successful EUS-guided PDD for benign PJAS between May 2011 and June 2025. The primary outcome was cumulative clinical fistula patency assessed by Kaplan–Meier analysis. Secondary outcomes included adverse events and risk factors for re-intervention. **Results:** Technical success was 95.83% (46/48), with clinical success in all technically successful cases. Cumulative clinical fistula patency rates were 82.20% at 1 year and 75.90% at 3 and 5 years. Postprocedural adverse events occurred in 28.26%. Unplanned re-intervention was required in 19.57%, exclusively among patients symptomatic before the initial procedure; symptomatic-status effects were therefore non-estimable. No independent predictors of unplanned re-intervention were detected, and no differences were observed between EUS-guided pancreaticogastrostomy (EUS-guided PGS) and EUS-guided pancreatojejunostomy (EUS-guided PJS). **Conclusions:** EUS-guided PDD achieved high technical and clinical success with durable long-term patency (82.20% at 1 year; 75.90% at 3 and 5 years). Differences between EUS-guided PGS and EUS-guided PJS and independent predictors of unplanned re-intervention were not detected; because all nine re-interventions occurred in patients symptomatic at baseline and none in asymptomatic patients, symptomatic-status effects were non-estimable.

## 1. Introduction

Pancreaticoduodenectomy is a complex and high-risk surgical procedure [1]. One of the notable long-term complications following pancreaticoduodenectomy is pancreaticojejunostomy anastomotic stricture (PJAS), which remains a significant clinical issue post-pancreaticobiliary surgery [2]. Murakami et al. reported that pancreatic duct dilatation caused by PJAS or obstruction occurred in 33.3% of pancreaticoduodenectomy patients one year after the operation [3]. The cause of PJAS has yet to be clearly identified [4]. The clinical manifestations of PJAS varied among patients, with postprandial abdominal pain and recurrent episodes of acute pancreatitis being the most frequently observed, followed by deterioration of pancreatic function [5]. Endoscopic retrograde pancreatography or EUS-guided pancreatic duct drainage (EUS-guided PDD) are commonly the first-line treatments for symptomatic PJAS after pancreaticoduodenectomy [2]. EUS-guided PDD is a valuable alternative when transpapillary approaches fail. It is effective in managing symptomatic obstructive pancreatitis, ductal strictures, or leakage by relieving pain and preserving both exocrine and endocrine pancreatic functions. In cases of tight anastomotic strictures, obstructing stones, or surgically altered anatomy, transpapillary drainage may not be feasible [6].

EUS-guided interventions are generally divided into drainage and rendezvous techniques [7]. Drainage can be achieved via the gastric route (EUS-guided pancreaticogastrostomy (EUS-guided PGS)) or jejunal routes (EUS-guided pancreatojejunostomy (EUS-guided PJS)) [8]. Postsurgical reconstruction often makes it highly challenging to advance a conventional endoscope to the PJAS site [4]. The majority of EUS-guided PDD procedures are performed for benign conditions, necessitating long-term stent maintenance and regular exchanges [9]. Despite the increasing adoption of EUS-guided PDD for the management of benign PJAS, data on its long-term outcomes and the risk factors associated with unplanned re-intervention remain limited. Our primary objective was to evaluate long-term cumulative clinical fistula patency after EUS-guided PDD; secondary objectives were to explore predictors of re-intervention. In this single-center cohort, EUS-guided PDD showed high technical and clinical success with durable patency over 5 years. We hypothesized that selected baseline and technical factors are associated with cumulative clinical patency and the risk of unplanned re-intervention. To explicitly state the novelty, this study provides a multi-year, single-center evaluation focused on benign PJAS and applies a pre-specified, protocolized stent-maintenance strategy, a fully covered self-expandable metal stent (FCSEMS) with a parallel 7 Fr plastic stent exchanged every three months and removed at one year, across the period 13 May 2011 to 30 June 2025.

## 2. Patients and Methods

### 2.1. Study Design and Setting

This single-center retrospective cohort study included data collected from 13 May 2011 to 30 June 2025. All initial EUS-guided PDD were performed at Aichi Cancer Center Hospital, Japan.

### 2.2. Patient Selection

#### 2.2.1. Inclusion Criteria

Patients were included based on the following criteria: patients aged over 18 years; a history of pancreaticoduodenectomy; a confirmed diagnosis of benign PJAS based on imaging or EUS examination; having undergone EUS-guided PGS or EUS-guided PJS; and the ability to complete a 1-year follow-up period after the initial EUS-guided PDD procedure.

#### 2.2.2. Exclusion Criteria

Exclusion criteria included patients with a follow-up duration of less than one year after initial EUS-guided PDD; and those in whom EUS-guided PGS or EUS-guided PJS could not be completed through successful stent deployment.

### 2.3. Endoscopic Ultrasound-Guided Pancreatic Duct Drainage Procedure

**Figure 1:**
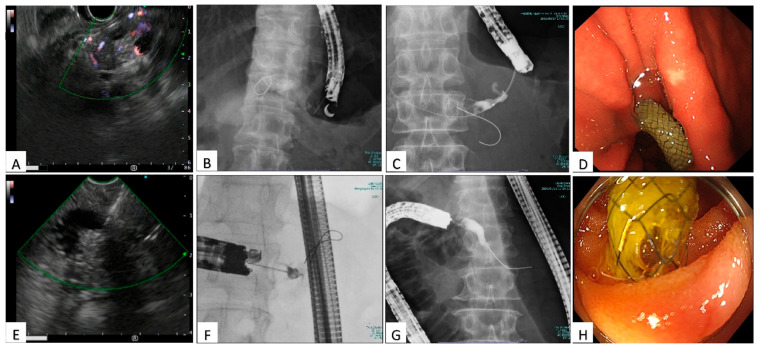
Stepwise images of EUS-guided PDD procedures. (**A**,**B**) Echoendoscope positioning and main pancreatic duct (MPD) puncture for PGS; (**C**,**D**) Guidewire advancement and stent deployment for PGS; (**E**,**F**) Echoendoscope positioning and puncture for PJS; (**G**,**H**) Guidewire placement and stent deployment for PJS.

For EUS-guided PGS, the pancreatic duct was identified using a linear echoendoscope (GF-UCT260; Olympus Medical Systems, Tokyo, Japan). For EUS-guided PJS, a forward-viewing echoendoscope (TGF-UCT260J; Olympus Medical Systems, Tokyo, Japan) was advanced to the anastomotic region to visualize the duct. The stepwise procedure of EUS-guided PDD is illustrated in Figure 1. Duct puncture was performed using either a 22-gauge needle (Expect Slimline; Boston Scientific, Marlborough, MA, USA) with a 0.018-inch guidewire (Fielder; Asahi Intecc, Seto, Aichi, Japan) or a 19-gauge needle (EZ Shot 3 Plus; Olympus Medical Systems, Tokyo, Japan) with a 0.025-inch guidewire (M-Through (Asahi Intecc, Seto, Aichi, Japan) or VisiGlide 2 (Olympus Medical Systems, Tokyo, Japan)), selected based on duct diameter and access route. In EUS-guided PJS, puncture was typically performed at the anastomotic entry site to avoid trans-parenchymal access. In both approaches, Doppler mode was used to avoid intervening vasculature. After successful puncture, the guidewire was advanced across the stricture, and the tract was dilated using coaxial-type electrocautery dilators (Fine 025 (Medico’s Hirata, Osaka, Japan) or Cysto-Gastro Set (ENDO-FLEX, Voerde, Germany)). Further dilation, if necessary, was performed with a 7 Fr ES dilator (Zeon Medical, Tokyo, Japan) or a 4–6 mm balloon catheter (REN; Kaneka Medix, Osaka, Japan). A 6 Fr uneven double-lumen cannula (Piolax Medical Devices, Yokohama, Japan) or a 5.5 Fr Tandem XL triple-lumen cannula (Boston Scientific, Marlborough, MA, USA) was used to facilitate contrast injection and guidewire exchange to a 0.025-inch VisiGlide 2 or M-Through guidewire. In most cases, a 6 mm × 12 cm FCSEMS HANAROSTENT^®^ Biliary Full Cover Benefit™ (M.I. Tech, Pyeongtaek, Republic of Korea) was used for EUS-guided PGS, whereas a 6 mm × 6 cm FCSEMS HANAROSTENT^®^ Biliary Full Cover Benefit™ was typically selected for EUS-guided PJS. This technique aligns with previously published methods at our institution [10,11]. Following initial stent placement, a 7 Fr plastic stent was routinely inserted in parallel and exchanged every three months. Plastic stents were removed after one year, followed by a period of observation.

In addition to the HANAROSTENT^®^ Biliary Full Cover Benefit™, other FCSEMS types employed in this study included the Niti-S™ Biliary S-type (Taewoong Medical, Gimpo, Republic of Korea) and HANAROSTENT^®^ Biliary Full Cover (M.I. Tech, Pyeongtaek, Republic of Korea). Plastic stents used included Through & Pass^®^ and Through & Pass^®^ TYPE IT (Gadelius Medical, Tokyo, Japan), Flexima™ Biliary Stent and Advanix™ Pancreatic Stent (Boston Scientific, Marlborough, MA, USA), and CATHEX™ Stick-shaped Pancreatic Stent (Cathex, Tokyo, Japan).

### 2.4. Definitions

The occurrence of benign PJAS was identified based on imaging modalities such as abdominal computed tomography, magnetic resonance cholangiopancreatography, abdominal magnetic resonance imaging, or EUS, which were performed regularly for each patient. These examinations revealed noticeable changes in the pancreatic duct, including early mild dilation or even significant dilation and progressive ductal deterioration, thereby allowing detection of strictures prior to symptom development.

Initial EUS-guided PDD was defined as the first EUS-guided PGS or EUS-guided PJS performed for each patient in which successful stent deployment was achieved and effective pancreatic drainage was established.

Symptoms were defined as clinically significant manifestations that led to medical consultation or prior hospitalization. Asymptomatic patients were those who did not exhibit any of these symptoms.

MPD diameter prior to EUS-guided PDD was measured as the largest diameter identified on preprocedural imaging modalities.

The criteria for selecting EUS-guided PJS or EUS-guided PGS largely depend on the accessibility of the anastomotic site using enteroscopy. If the anastomotic site can be reached, EUS-guided PJS is preferred. Otherwise, EUS-guided PGS is chosen.

Postprocedural adverse events were defined and graded according to the American Society for Gastrointestinal Endoscopy lexicon.

Unplanned re-intervention was defined as any subsequent procedure involving either repeat puncture or the creation of a new fistulous tract using EUS-guided PGS or EUS-guided PJS, as well as re-dilation of an existing fistula using a dilator. The procedure was considered a re-intervention only when it was completed with successful stent deployment and effective pancreatic drainage.

Technical success was defined as achieving all of the following in a single session: successful puncture of the pancreatic duct, establishment of drainage, and successful stent deployment.

Clinical success was defined as the absence of the need for re-intervention within 90 days following the initial EUS-guided PDD. This definition is consistent with recent international reports on EUS-guided PDD outcomes.

### 2.5. Endpoints

The primary outcome was the cumulative clinical fistula patency after EUS-guided PDD, defined as the duration from the index procedure to the first re-intervention. Secondary outcomes included the technical and clinical success rates of EUS-guided PDD, the rate and severity of adverse events, and the incidence of unplanned re-intervention. Long-term outcomes were further evaluated by analyzing associated variables, including the interval from pancreaticoduodenectomy to initial EUS-guided PDD, preprocedural symptom status, MPD diameter, type of EUS-guided PDD procedure, stent type, use of electrocautery dilators, and postprocedural adverse events.

### 2.6. Statistical Analysis

SPSS version 23.0 (IBM, Armonk, NY, USA) was used for all statistical analyses. Continuous variables were summarized as medians and ranges, and categorical variables were presented as frequencies and percentages.

Analyses of clinical outcomes, adverse events, long-term patency, and re-intervention were limited to patients who achieved technical success (*n* = 46). Cumulative clinical fistula patency was evaluated using the Kaplan–Meier method. The primary outcome was cumulative clinical fistula patency, defined as the time from the initial EUS-guided PDD procedure (index procedure) to the first unplanned re-intervention related to recurrence of PJAS. Patients without re-intervention were censored at the date of last follow-up. The median patency duration and cumulative patency rates at 12, 36, and 60 months were calculated.

Risk factors for unplanned re-intervention were explored using univariable and multivariable Cox proportional hazards models. Variables with a *p*-value < 0.20 in univariate analysis were included in the multivariate model. Hazard ratios (HRs) and 95% confidence intervals (CIs) were reported, and statistical significance was defined as a two-sided *p*-value < 0.05. Because re-intervention events were sparse overall (including only one event in the EUS-guided PJS group), fixed-time Kaplan–Meier comparisons between EUS-guided PGS and EUS-guided PJS were considered unstable; therefore, group differences were summarized using Cox proportional hazards models.

## 3. Results

**Figure 2:**
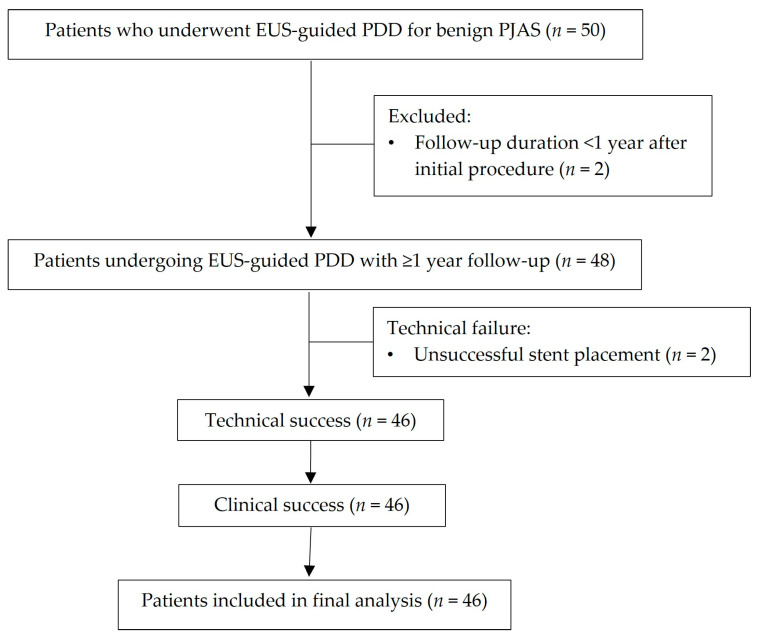
Flowchart of patient selection and inclusion for final analysis.

Among the 50 patients who underwent EUS-guided PDD, two were excluded because their follow-up period was less than one year. The remaining 48 patients were evaluable, and technical success was achieved in 46 of them; these 46 patients constituted the final study cohort (Figure 2).

### 3.1. Patient Demographics and Baseline Clinical Characteristics

Patient demographics, indications for surgery, and causes of ductal obstruction are summarized in Table 1.

### 3.2. Procedural Success and Adverse Events

Technical success was achieved in 46 of 48 attempted procedures (95.83%). All patients with technical success (46/46) achieved clinical success. Symptomatic presentation prior to EUS-guided PDD included abdominal pain (46.88%), pancreatitis (18.75%), diarrhea (15.63%), fever (9.38%), back pain (6.25%), and cholangitis (3.13%). Postprocedural adverse events occurred in 28.26% (13/46) of patients. Among them, 23.08% (3/13) were mild, 69.23% (9/13) moderate, and 7.69% (1/13) severe.

### 3.3. Long-Term Outcomes

Unplanned re-intervention occurred in 19.57% of patients, all of whom had been symptomatic prior to the initial procedure. Of those needing re-intervention, 8 had undergone EUS-guided PGS and 1 had received EUS-guided PJS. The detailed patient outcomes are summarized in Table 2.

**Figure 3:**
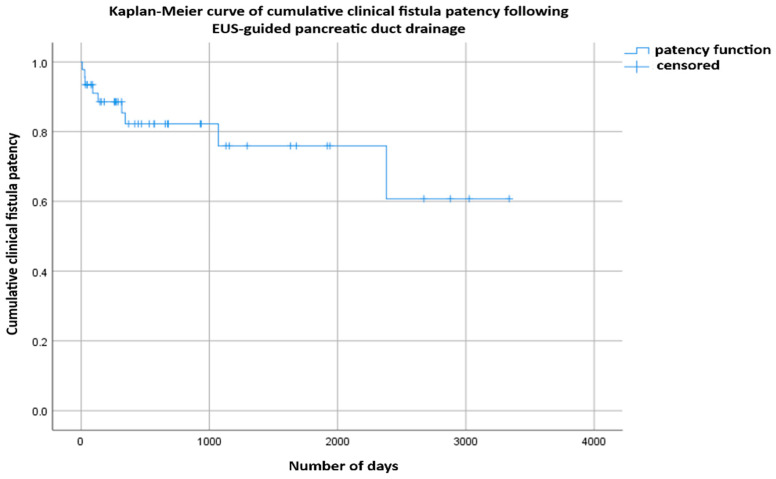
Kaplan–Meier curve showing cumulative clinical fistula patency following EUS-guided PDD. Patency was defined as the absence of re-intervention. Time-to-event analysis included patients who became stent-free and those with long-term stent exchange. Crosses (+) indicate censored cases.

Kaplan–Meier analysis demonstrated cumulative clinical fistula patency rates of 82.20% at 12 months and 75.90% at both 36 and 60 months (Figure 3). The median (50%) patency duration was not reached during follow-up, indicating sustained long-term efficacy after EUS-guided PDD in patients with benign PJAS. These findings suggest that EUS-guided PDD provides durable long-term drainage in most patients, with a 5-year cumulative patency rate of 75.90%.

### 3.4. Risk Factor Analysis

Risk factors for unplanned re-intervention were assessed using univariate and multivariate Cox proportional hazards models (Table 3). No statistically significant predictors of unplanned re-intervention were identified on multivariable analysis.

## 4. Discussion

In this study, we aimed to evaluate the long-term outcomes and potential predictors for re-intervention following EUS-guided PDD in patients with benign PJAS. The procedure demonstrated high technical (95.83%) and clinical (100.00%) success rates. Most patients remained free from re-intervention, with a 1-year clinical fistula patency rate of 82.20% and 3- and 5-year rates of 75.90%. The median cumulative clinical fistula patency was not reached during the follow-up period, suggesting that EUS-guided PDD provides durable long-term drainage in most patients. The minimal decline in patency between the first and fifth years implies that once initial drainage is successfully established, the need for re-intervention remains low over time.

EUS-guided PDD was selectively performed in patients with well-defined clinical indications.

With regard to procedural indications, the decision to perform EUS-guided PDD was not based solely on imaging findings but was instead made on a case-by-case basis. The benign PJAS observed in this cohort typically represents a slowly progressive, fibrotic stricture at the anastomotic site. This fibrosis may gradually evolve over time and take years to result in clinically significant obstruction or symptoms. In many cases, strictures had already been present for a considerable duration but remained asymptomatic, without overt clinical manifestations or laboratory abnormalities. Patients were monitored closely, and intervention was reserved for those in whom it was deemed necessary.

Notably, all re-intervention cases occurred exclusively in symptomatic patients prior to initial EUS-guided PDD. This finding may be explained by the underlying pathophysiology of pain in benign pancreatic duct obstruction, which is commonly attributed to ductal hypertension secondary to luminal obstruction [12]. Preprocedural symptoms may indicate more advanced or clinically significant stricture or persistent inflammatory changes at the anastomotic site. These factors could contribute to stent dysfunction and thereby increase the likelihood of re-intervention. Therefore, the association between preprocedural symptoms and re-intervention observed in our study likely reflects both the nature of the stricture and the pragmatic nature of real-world clinical practice.

EUS-guided PGS was more commonly performed than EUS-guided PJS due to anatomical limitations in reaching the anastomotic site. Although EUS-guided PJS is technically feasible, advancing a guidewire across a stenosed anastomosis can be particularly challenging. As a result, EUS-guided PGS remains widely used in patients with surgically altered anatomy, offering more accessible routes and facilitating potential re-interventions [13]. Ultimately, the therapeutic goal in managing benign PJAS is to achieve complete stricture resolution, allowing for stent removal [6].

In this study, most unplanned re-intervention events occurred in the EUS-guided PGS group, though the difference was not statistically significant. Our findings suggest that unplanned re-intervention is multifactorial and not solely dependent on technical or anatomical variables. EUS-guided PJS may provide improved long-term patency through the creation of a new drainage route, thereby reducing the need for repeat interventions [13]. This is consistent with findings by Sadek et al., who demonstrated that EUS-guided PJS is a promising and safe technique for the management of PJAS after pancreaticoduodenectomy. This technique offers potential advantages, such as a reduced rate of re-interventions and the formation of a stable, long-lasting drainage tract [11]. Stent dysfunction is particularly common in patients undergoing EUS-guided PGS, with reported dysfunction rates reaching up to 55% [14]. This aligns with our data, in which 19.57% (9/46) of patients required re-intervention. Among them, 88.89% had undergone EUS-guided PGS, while only one had received EUS-guided PJS. Although this numerical difference suggests a potential trend toward a higher re-intervention rate in the EUS-guided PGS group, it was not statistically significant (*p* = 0.98). Consistent with prior reports, EUS-guided PJS may offer more durable outcomes due to its physiological alignment and reduced risk of stent migration.

Electrocautery dilators were used in 41.30% of cases. The degree of difficulty and the need for tract dilation may vary depending on the firmness of the pancreatic tissue. When the pancreas is notably firm or ‘hard,’ forceful tract dilation is often required, and the use of electrocautery dilators may be warranted [15]. Electrocautery dilators are typically reserved for cases where the stricture is too tight or fibrotic for mechanical or balloon dilators to traverse, reflecting a technically challenging scenario. Thermal injuries and subsequent inflammation or fibrosis may compromise long-term tract integrity and contribute to stent dysfunction or the need for early re-intervention. Although not a significant predictor of re-intervention, their use may indicate higher procedural complexity and increased risk of thermal injury, which could compromise tract integrity and stent function.

Given the event sparsity and group imbalance, fixed-time Kaplan–Meier contrasts were not emphasized; we relied on Cox modeling for more stable inference. However, symptomatic status and electrocautery dilator use showed non-significant trends toward increased risk. The wide confidence intervals reflect the limited power of our sample, but they also underscore the multifactorial nature of stent failure.

All cases of re-intervention occurred in patients who had been symptomatic prior to their initial procedure, suggesting that preprocedural symptoms may reflect a higher-risk subgroup with more advanced disease or inflammatory burden. Thus, symptomatic patients may harbor more advanced or functionally significant strictures. This could explain their higher likelihood of requiring re-intervention. Our findings support the practice of tailoring intervention timing based on both imaging and symptom evolution.

In this study, EUS-guided PDD failed in two patients. The main challenges included accessing the pancreatojejunostomy loop, identifying the anastomotic site, and successfully cannulating the MPD, especially in cases with severe stricture [4]. In such cases, if guidewire stability under EUS visualization cannot be maintained, placement of a transmural stent may not be feasible [6]. These procedural challenges may have contributed to the failed attempts in our study.

This study possesses several notable strengths, including a relatively long duration of follow-up, standardized procedural techniques performed at a high-volume tertiary referral center, and a clear procedural distinction between EUS-guided PGS and EUS-guided PJS. Moreover, the use of clearly defined clinical endpoints and adverse events criteria, based on established classification systems, enhances the reproducibility and clinical applicability of our findings. Nonetheless, several limitations should be acknowledged. As a retrospective, single-center study, the findings are inherently subject to selection bias. The relatively low number of re-intervention events limited the statistical power to detect significant associations in multivariate analysis. Future studies should adopt a prospective, multicenter design with larger sample sizes and standardized outcome definitions to validate the long-term efficacy and safety of EUS-guided PDD for benign PJAS.

## 5. Conclusions

EUS-guided PDD achieved high technical and clinical success with durable long-term patency (82.20% at 1 year; 75.90% at 3 and 5 years). Differences between EUS-guided PGS and EUS-guided PJS and independent predictors of unplanned re-intervention were not detected; because all nine re-interventions occurred in patients symptomatic at baseline and none in asymptomatic patients, symptomatic-status effects were non-estimable.

## Figures and Tables

**Table 1:** Baseline patient characteristics.

Variable	EUS-Guided PDD (*n* = 46)
Age (years)	75.50
Gender	
Female	23 (50%)
Male	23 (50%)
Indications for PD	
IPMN	14 (30.43%)
Pancreatic carcinoma	11 (23.91%)
IPMC	4 (8.70%)
Pancreatic NET	4 (8.70%)
Bile duct carcinoma	4 (8.70%)
Vater carcinoma	2 (4.35%)
Duodenal GIST	2 (4.35%)
Carcinoma of duodenal papilla	1 (2.17%)
MCN	1 (2.17%)
SCN	1 (2.17%)
SPN	1 (2.17%)
Vater NET	1 (2.17%)
Etiology of ductal obstruction leading to EUS-guided PDD	
Pancreatic stone	23 (50%)
Pancreatitis	5 (10.87%)
Increased amylase	3 (6.52%)
Pancreatic duct debris	3 (6.52%)
Pseudocyst	3 (6.52%)
Others	9 (19.57%)

EUS-guided PDD, endoscopic ultrasound-guided pancreatic duct drainage; PD, pancreaticoduodenectomy; IPMN, intraductal papillary mucinous neoplasms; IPMC, intraductal papillary mucinous carcinoma; NET, neuroendocrine tumor; GIST, gastrointestinal stromal tumor; MCN, mucinous cystic neoplasm; SCN, serous cystic neoplasm; SPN, solid pseudopapillary neoplasm.

**Table 2:** Patient outcomes.

Variable	Number of Patients (*n* = 46)
Interval between PD and development of PJAS	
≥478.50 days	23 (50%)
<478.50 days	23 (50%)
Interval between PD and initial EUS-guided PDD	
≥1145.50 days	23 (50%)
<1145.50 days	23 (50%)
Preprocedural Symptom Status	
Asymptomatic	14 (30.43%)
Symptomatic	32 (69.57%)
MPD diameter prior to EUS-guided PDD	
≥5 mm	14 (30.43%)
<5 mm	32 (69.57%)
EUS-guided PDD	
EUS-guided PGS	34 (73.91%)
EUS-guided PJS	12 (26.09%)
The type of stent	
Metal stent	27 (58.70%)
Plastic stent	19 (41.30%)
The use of electrocautery dilator	
Yes	19 (41.30%)
No	27 (58.70%)
Post-EUS-guided PDD adverse events	
Yes	13 (28.26%)
No	33 (71.74%)
Patients with unplanned re-intervention	
Yes	9 (19.57%)
No	37 (80.43%)

PD, pancreaticoduodenectomy; PJAS, pancreaticojejunostomy anastomotic stricture; EUS-guided PDD, endoscopic ultrasound-guided pancreatic duct drainage; EUS-guided PGS, endoscopic ultrasound-guided pancreaticogastrostomy; EUS-guided PJS, endoscopic ultrasound-guided pancreatojejunostomy.

**Table 3:** Risk factors for re-intervention after EUS-guided PDD.

Variable	Univariate HR (95% CI)	*p*-Value	Multivariate HR (95% CI)	*p*-Value
Interval between PD and initial EUS-guided PDD	1.21 (0.32–4.54)	0.78	1.41 (0.36–5.54)	0.62
Symptomatic vs. asymptomatic	Not applicable *	-	-	-
MPD ≥ 5 mm	0.59 (0.12–2.84)	0.51	0.63 (0.11–3.53)	0.60
EUS-guided PGS	1.73 (0.20–14.7)	0.61	1.04 (0.08–12.9)	0.98
Metal stent	1.12 (0.28–4.53)	0.87	0.68 (0.10–4.75)	0.70
Electrocautery dilator	1.73 (0.40–7.45)	0.46	3.15 (0.36–27.9)	0.30
Post-EUS-guided PDD adverse events	0.24 (0.03–1.96)	0.18	0.39 (0.04–3.77)	0.42

(*) Symptomatic status was not analyzed due to the absence of re-intervention in asymptomatic patients. EUS-guided PDD, endoscopic ultrasound-guided pancreatic duct drainage; HR, hazard ratio; CI, confidence interval; PD, pancreaticoduodenectomy; MPD, main pancreatic duct; EUS-guided PGS, endoscopic ultrasound-guided pancreaticogastrostomy.

## Data Availability

No new data were created or analyzed in this study.
